# Superior visible light antimicrobial performance of facet engineered cobalt doped TiO_2_ mesocrystals in pathogenic bacterium and fungi

**DOI:** 10.1038/s41598-021-84989-x

**Published:** 2021-03-10

**Authors:** Ayat N. El-Shazly, Gharieb S. El-Sayyad, Aiat H. Hegazy, Mahmoud A. Hamza, Rasha M. Fathy, E. T. El Shenawy, Nageh K. Allam

**Affiliations:** 1 Energy Materials Laboratory, School of Sciences and Engineering, The American University in Cairo, New Cairo, 11835 Egypt; 2 Central Metallurgical Research and Development Institute, Helwan, P.O. Box 87, Cairo, Egypt; 3 Drug Microbiology Lab., Drug Radiation Research Department, National Center for Radiation Research and Technology (NCRRT), Egyptian Atomic Energy Authority (EAEA), Cairo, Egypt; 4 Solar Energy Department, National Research Centre, Giza, Dokki Egypt; 5 Chemistry Department, Faculty of Science, Ain Shams University, Abbassia, Cairo, Egypt

**Keywords:** Pathogenesis, Engineering, Nanoscience and technology

## Abstract

Pristine and Co-doped TiO_2_ mesocrystals have been synthesized via a simple sol–gel method and their antimicrobial activity has been investigated. The antimicrobial performance was evaluated in terms of zone of inhibition, minimum inhibitory concentration (MIC), antibiofilm activity, and effect of UV illumination in liquid media. The Co-doped TiO_2_ mesocrystals showed very promising MIC of 0.390 μg/mL and 0.781 μg/mL for *P. mirabilis* and *P. mirabilis*, respectively*.* Additionally, the material showed an MIC of 12.5 μg/mL against *C. albicans*, suggesting its use as antifungal agent. Upon the addition of 10.0 µg/mL of Co-doped TiO_2_ mesocrystals, the biofilm inhibition% reaches 84.43% for *P. aeruginosa*, 78.58% for *P. mirabilis*, and 77.81% for *S. typhi*, which can be ascribed to the created active oxygen species that decompose the tested microbial cells upon illumination. Thus the fabricated Co-doped TiO_2_ mesocrystals exhibit sufficient antimicrobial features under visible light, qualifying them for use as antimicrobial agents against pathogenic bacteria and fungi and subsequently inhibit their hazardous effects.

## Introduction

In the last decades, a lot of efforts have been devoted towards the synthesis of nanomaterials with unique physical, chemical, and biological characteristics compared to their bulk counterparts^1,2^. Cölfen et al. first introduced a new class of materials known as mesocrystal^3^. Mesocrystals were proposed to form upon the addition of highly oriented small particles, thus the resulting larger crystals would have single-crystal orientation^4^. Their positive effects in improving charge carriers separation made them good candidates for many applications, such as photocatalysis^5^, sensing, and energy storage and conversion^6^. For instance, TiO_2_ mesocrystals become a research hotspot for biomedical and food applications due to their antimicrobial characteristics^7^. Besides, their chemical stability, abundance, low cost, eco-friendly made them good candidates for photovoltaics^8^, hydrogen production^9^ and wastewater treatment^10^. Upon irradiated by UV light, the anatase phase of TiO_2_ can oxidize and reduce oxygen and water to produce reactive oxygen species (ROS), such as superoxide radicals and hydroxyl radicals^11^. These ROS play a key role in destroying pathogenic bacteria and fungi by damaging their critical molecular components^12,13^. However, the practical application of TiO_2_ photocatalysts is limited by their wide bandgap energy (3.0–3.2 eV), limited to the UV region of the light spectrum with low efficiency of solar light energy utilization^14–18^. To enhance the photocatalytic response of TiO_2_, many strategies have been implemented, such as metal and nonmetal doping, annealing in reducing atmosphere, creating defects in the crystal lattice, and coupling with various light harvesters^16,19,20^. Doping TiO_2_ with transition metals, such as Fe, Ni, Cr, and Co, was shown to enhance its photoactivity by creating shallow states that suppress the e–h pairs recombination^21,22^. Moreover, multi-doping with two or three metal or non-metal elements, such as N, C, and Ce or Co, Cu, Ir, C, and Ti^3+^, was shown to improve the conductivity and balance the deficiencies of individual dopants^23,24^. Consequently, multi-doped TiO_2_ has gained much attention compared to the singly and doubly-doped counterparts. The origin of the super reactivity of multi-doped TiO_2_ seems to be the synergistic effect of the dopants in narrowing the bandgap, enhancing the concentration of reactive radical species^25^, and enhancing visible light absorption^24^. In this regard, identifying a one-step synthesis method of multi-doped TiO_2_ is extremely desirable, which remains a challenge to be realized. Herein, we report on the successful fabrication of cobalt, Ti^3+^, and carbon multi-doped TiO_2_ mesocrystals via an in-situ sol–gel process. The antimicrobial behavior of the fabricated mesocrystals was investigated at ambient conditions and under light illumination. Finally, the reaction mechanism of TiO_2_ and multi-doped TiO_2_ mesocrystals-treated microbial cells was suggested and discussed in details. Thus, the innovative points of this research include the one-pot synthesis with controlled amount of dopants, the defective structures and how defects played a role in the antibacterial properties as well as the superior dual bacterial and fungi inhibition functions.

## Materials and methods

Titanium n-propoxide (Ti(O-n-Pr)_4_, 98%), cobalt nitrate hexahydrate (Co (NO_3_)_2_·6H_2_O), Formamide (FA: H_2_N-CHO), and hydrofluoric acid (HF 40%) were purchased from Sigma-Aldrich. Microbiological media ingredients were purchased from Oxford, and reagents used in the biological tests were obtained from Sigma-Aldrich. All the other chemicals were of pure grade and used as received without any further purification. All the solutions were prepared using distilled water (DW).

### Synthesis of TiO_2_ mesocrystals

The proposed TiO_2_ mesocrystals were fabricated using a facile one-pot synthesis method, inspired by the work reported by Hegazy and Prouzet^26^. Typically, 3 mL of HF was added dropwise to 4.84 mL of Ti(O-n-Pr)_4_ with vigorous stirring in an ice bath. Then, 5.2 mL of a solution of FA in DW (86%, v/v) were added dropwise to the previous solution, which was left for 2 h at room temperature. The resulting gel was dried at 100 °C for 4 h and calcined in air at 400 °C for 4 h^18^. The Co-doped TiO_2_ mesocrystals were synthesized according to the same procedure by dissolving 0.5 g (Co(NO_3_)_2_) in the mixture of FA/DW.

### Physicochemical characterizations and antimicrobial activities of TiO_2_ and Co-doped TiO_2_ mesocrystals

The crystal properties of the as-synthesized samples were investigated by X-ray diffraction (XRD) patterns recorded on PANalytical X’Pert PRO X-ray diffractometer with Cu Kα radiation (λ = 0.15418 nm, 2θ range = 5°:80°, step size = 0.04°, and scan-step time = 0.5 s). Raman measurements were performed on a Raman microscope (Pro Raman-L Analyzer) with an excitation laser beam wavelength of 532 nm. Fourier transform infrared (FTIR) spectra were recorded on Nicolet 380 Thermo-Scientific in the range of 400–4000 cm^−1^. The elemental composition was assessed using Thermo-Scientific ESCALAB 250Xi X-ray photoelectron spectroscopy (XPS). The morphological analysis of the as-synthesized nanoparticles was performed using a Zeiss SEM Ultra 60 field-emission scanning electron microscope (FESEM) operating at an accelerating voltage of 5 kV. The nanostructure of the samples was investigated using JOEL JEM-2100 high-resolution transmission electron microscope (HR-TEM) operating at an accelerating voltage of 200 kV; the sample was prepared by dispersing the TiO_2_ powder in ethanol followed by dropping a small amount on a standard copper TEM grid containing lacy carbon. The UV–Vis absorption spectra of samples were collected using a Shimadzu UV-2600 UV–Vis–NIR spectrophotometer. The photoluminescence spectra (PL) were recorded using Thermo- Scientific LUMINA fluorescence spectrometer.

The antimicrobial potential of as-synthesized TiO_2_ mesocrystals, Co-doped TiO_2_ mesocrystals, and Co^2+^ ions against different pathogenic microbes (yeast and bacteria) are examined via employing the agar-disc diffusion method^12^. Firstly, the as-synthesized TiO_2_ mesocrystals, and Co^2+^ ions are dissolved into distilled water with concentrations 0.01 mg/mL; 10 ppm. The activity of the as-synthesized compounds are examined against different types of bacteria, namely *Staphylococcus aureus*, *Pseudomonas aeruginosa, Escherichia coli*, *Klebsiella pneumoniae*, Methicillin-resistant *Staphylococcus aureus* (MRSA), *Proteus vulgaris*, *Salmonella typhi,* and *Proteus mirabilis.* The examined multi-drug resistance bacteria were tested by Vitek two systems (bioMarieux and Marcy-LEtoile, France). Most of them were resistant to antibiotics like Cefapirin, Ciprofloxacin, Amikacin, Norfloxacin, Amoxicillin, Cefoxitin, Gentamicin, Ampicillin, and Cefotaxime. In the microbiological experiments, we performed the biosafety Level-2 (BSL-2). It should be noted that all the inoculums are established and fixed from 2–5 × 10^8^ CFU/mL (0.5 McFarland; at 600 nm). The inhibition of the bacterial growth was defined by the zone of inhibition (ZOI) after 24 h of incubation. Additionally, the antifungal potential of the as-synthesized TiO_2_ mesocrystals, and Co^2+^ ions is examined against pathogenic unicellular fungi (*Candida albicans* and *Candida tropicalic*). After that, the inoculums of the tested yeast cells are set from 1–4 × 10^7^ CFU/mL. Finally, Nystatin (NS) and Amoxicillin (AX) are conducted as standard antibiotics. AX is similar to penicillin in its bactericidal action against susceptible bacteria during the stage of active multiplication. It acts via the inhibition of cell wall biosynthesis that leads to the death of the bacteria. While, NS is an antifungal that is both fungi-static and fungicidal in vitro against a wide-variety of yeasts and yeast-like fungi. It exerts its antifungal effects via disruption of the fungal cell membrane.

The minimum inhibitory concentrations (MIC) investigation is completed in Luria–Bertani (LB) broth within a serial dilution. Briefly, a positive control (the microorganism and the nutrient), a negative control (the nutrient solely), and the examined TiO_2_ mesocrystals (beginning with 0.1 mg/mL concentration; 100 ppm) are applied; MIC is defined following 24 h at 37 °C. The inoculums of the tested bacteria are at 3–5 × 10^8^ CFU/mL and 2–3 × 10^7^ CFU/mL to *Candida* species. MIC is defined by operating ELISA plate (at 600 nm). Finally, the results are statistically analyzed by applying ONE WAY ANOVA, the least significant difference (LSD), and Duncan's multiple ranges, which are calculated by special software (SPSS version 15).

### Antibiofilm activities of Co-doped TiO_2_ mesocrystals

Moreover, a qualitative measurement regarding the biofilm inhibition was defined as stated by G. Christensen et al.^27^. The noticeable examination of the biofilm which was performed at the tube wall in the absence and presence of the synthesized TiO_2_ mesocrystals was established. The antibiofilm of the as-synthesized TiO_2_ mesocrystals (at 10.0 µg/mL) was examined toward the selected bacteria and *Candida *spp., and was determined and compared with the control (non-treated one). Briefly, 5 mL of the nutrient broth medium was added inside all tubes, and the examined bacteria and yeast were inoculated after adjusted 0.5 McFarland to be 1–2.5 × 10^8^ CFU/mL. After that, they were incubated at 37.0 ± 0.5 °C for 24 h. The contents presented in control and treated tubes were discarded, mixed with Phosphate Buffer Saline (PBS; pH 7.0), and finally desiccated. Then, the bacterial and yeast cells which adhered to the tube walls were fixed with 5 mL sodium acetate (3.0%) for about 15 min, and finally, they were rinsed with de-ionized water. Biofilms which introduced inside tubes were stained with 15 mL Crystal Violet (CV; 0.1%) and washed with de-ionized water to remove the rest of the CV. It must be noted that, for the semi-quantitative antibiofilm estimation, 5 mL of the absolute ethanol was inserted to dissolve the stained bacterial and yeast biofilms^28–30^. The O.D. of the stained bacterial and yeast biofilms with CV was examined by UV–Vis. spectrophotometer at 570.0 nm. The bacterial and yeast biofilms inhibition percentage was estimated by applying the following relation (Eq. 1) ^31^:

1 $$ {\text{Biofilm }}\,{\text{inhibition }}\% = \left( {{\text{O}}.{\text{D}}._{{{\text{Control }}\,{\text{sample}}}} - {\text{O}}.{\text{D}}._{{{\text{treated }}\,{\text{sample}}}} } \right)/{\text{O}}.{\text{D}}._{{{\text{Control }}\,{\text{sample}}}} \times {1}00. $$

### Effect of UV-irradiation on the antimicrobial abilities of the prepared TiO_2_ mesocrystals, and Co-doped TiO_2_ mesocrystals

Furthermore, the antibacterial activity of the as-synthesized TiO_2_ nanoparticles with and without UV illumination was assessed against the tested pathogenic microbes *Pseudomonas aeruginosa*, *Staphylococcus aureus*, and *Candida albicans* strains using the optical density method^32^. The tested microorganisms were stimulated in nutrient broth (NB) overnight at 37 °C. Firstly, 0.5 mL of the overnight culture were inoculated to 5 mL NB tubes that adjusted after 2 h of incubation to standard 0.5 McFarland concentration that standardly equals 1.5 × 10^8^ CFU of bacteria and 0.400 equal (1 × 10^4^ cells/mL) of C. *albicans*. 100 µL of Co-doped TiO_2_ mesocrystals were added into the tubes and then incubated at 37 °C for 60 min. While tubes without Co-doped TiO_2_ mesocrystals were inoculated with bacteria and used as the positive control (subject to UV), tubes without UV illumination were used as the negative control. Typically, 10-W low-pressure mercury lamp was horizontally-placed on the laminar flow and employed as the UV-irradiation source, where 90% of the emitted irradiation was at the specific wavelength (600 nm for bacteria and 630 nm for the fungi). Finally, test tubes were subject to UV-irradiation for 1 h at a distance of about 61 cm. After the incubation, the turbidity of the medium was measured at λ of 600 nm for bacteria and 630 nm for the fungi.

### Reaction mechanism using SEM/EDX analysis of TiO_2_ mesocrystals, and Co-doped TiO_2_ mesocrystals-treated microbial cells

The sensitive bacterial cells (from the antibiofilm results) were cleaned with Physiological Buffer Saline (PBS) three-times and finally, fixed by 3.5% glutaraldehyde solution. The maintained microbial units were repeatedly-rinsed by PBS and regularly-dried with different concentrations of ethyl alcohol like 30, 50, 70, 90, and 100% for 15 min at 27 ± 2 °C. Following that, the prepared samples were fixed on an aluminum piece regarding SEM/EDX analysis. The morphological features of the control (non-treated *P. aeruginosa*), TiO_2_ mesocrystals, and Co-doped TiO_2_ mesocrystals-treated *P. aeruginosa* were examined by SEM/EDX investigation.

## Results and discussion

### Physicochemical characterization

**Figure 1:**
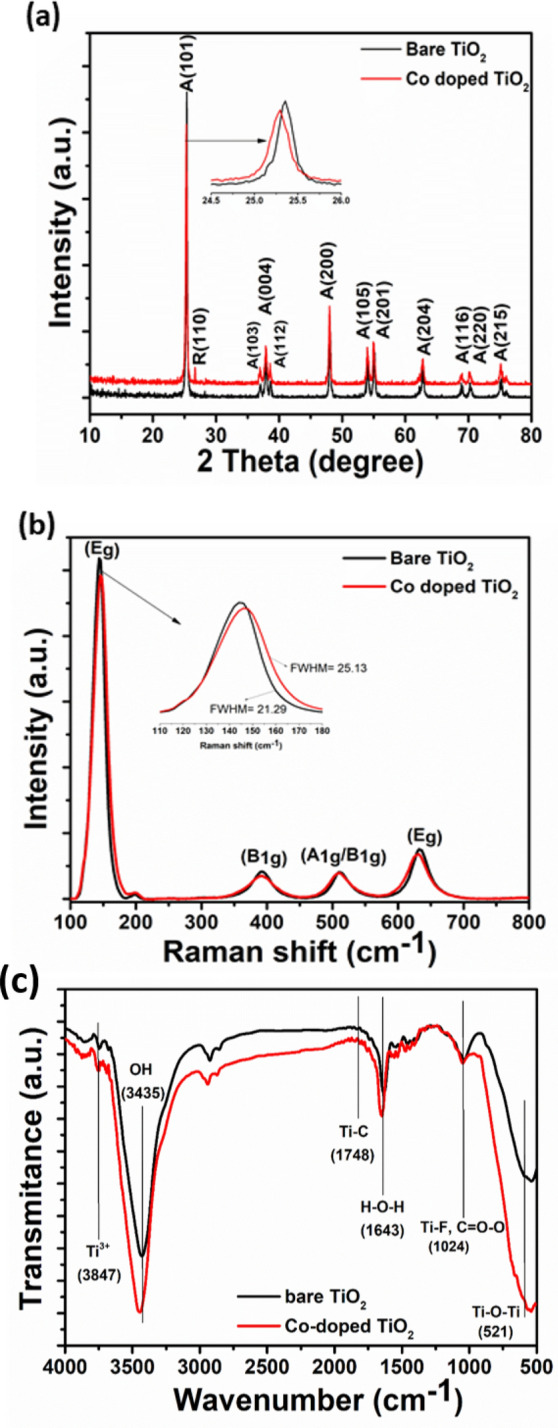
The (**a**) XRD patterns, (**b**) Raman, and (**c**) FT-IR spectra of the bare and Co-doped TiO_2_ mesocrystals.

Figure 1a depicts the XRD patterns of both bare and Co-doped TiO_2_ mesocrystals calcinated at 400 °C. Both samples have tetragonal anatase phase with a space group I41/amd (Ref card No.: 04-014-5762). No diffraction peaks for cobalt or cobalt oxide were detected in the Co-doped TiO_2_ sample, which may be related to the low cobalt content^33^. However, the main (1 0 1) diffraction peak is shifted to lower 2$\uptheta$ (inset in Fig. 1a), indicating the incorporation of foreign species into the TiO_2_ lattice, thus changing the Ti^4+^ local structure^34^. Besides, it was found that there is a low intensity peak at 26.7° in the diffraction pattern of the bare TiO_2_ sample, which can be attributed to the main plane (110) of the rutile phase. The intensity of this peak was enhanced after the insertion of cobalt ions as observed in the diffraction pattern of the Co-doped TiO_2_ sample. The percentage of both anatase and rutile phases was calculated using Eqs. (2) and (3)^20^:

2 $$ {\text{A}}\% \, = \, \left[ {0.{\text{79 I}}_{{\text{A}}} / \, \left( {{\text{I}}_{{\text{R}}} + \, 0.{\text{79I}}_{{\text{A}}} } \right)} \right] \, \times { 1}00, $$

3 $$ {\text{R}}\% \, = \, \left[ {{\text{ I}}_{{\text{R}}} / \, \left( {{\text{I}}_{{\text{R}}} + \, 0.{\text{79I}}_{{\text{A}}} } \right)} \right] \, \times { 1}00, $$

where I_A_ and I_R_ are the intensities of XRD peaks of anatase and rutile at 25.3° and 26.7° peaks, respectively. It was found that bare TiO_2_ sample is composed of 99% anatase and only 1% rutile, while the Co-doped TiO_2_ sample is composed of 93.3% anatase and 6.7% rutile. The increase of rutile% reveals that Co can act as a rutile stabilizer^35^. Although it is generally accepted for pure phases that anatase exhibits a higher photocatalytic activity compared to rutile TiO_2_, the existence of very small percent of the rutile phase causes an enhancement in the photocatalytic activity of the samples, even in the case of bare TiO_2_ mesocrystals, due to the synergistic effects between the two phases compared to pure phases^19^.

The mean crystallite sizes of bare TiO_2_ and Co-doped TiO_2_ were estimated to be 69 and 40 nm, respectively as determined using Debey–Scherrer formula (Eq. 4), with L being the mean crystallite size (nm), *k* the Scherrer constant related to the crystallite shape (k = 0.9), λ the X-ray wavelength in nanometer (nm), β the full width at half-maximum of the peak in radians, and θ the diffraction angle.

4 $$ L = \frac{k\lambda }{{\beta \cos \theta }}. $$

Note the decrease in crystallinity upon Co doping, which can be ascribed to the difference between the ionic charge of Ti (+4) and Co (+2). Moreover, Co doping is expected to cause a slight change in the lattice constants. The lattice parameters (*a* and *c*) and cell volume (*V*) were estimated based on Bragg’s law and a formula for a tetragonal system (Eq. 5) for both bare TiO_2_ and Co-doped TiO_2_ samples.

5 $$ \frac{1}{{d^{2} }} = \frac{{h^{2} + k^{2} }}{{a^{2} }} + \frac{{l^{2} }}{{c^{2} }}. $$

It was observed that the unit cell volume has been enlarged from 135.66 to 136.612, and the lattice constant “*a*” from 3.78 to 3.794 for bare TiO_2_ and Co-doped TiO_2_, respectively. These results may indicate the successful incorporation of Co^2+^ into the anatase TiO_2_ lattice.

Raman spectroscopy is an effective tool to elucidate the structural changes in materials upon doping. Based on group theory, anatase TiO_2_ exhibits six Raman-active vibrational modes (3 E_g_ + 2B_1g_ + A_1g_), as displayed in Fig. 1b. The six allowed modes of anatase single crystal were reported by Oshaka^36^, where the bands E_g1_ (at 144 cm^−1^), E_g2_ (at 196 cm^−1^), and E_g3_ (at 632 cm^−1^), for Eg modes are related to the O–Ti–O symmetric stretching vibration. The two B_1g_ modes: B_1g1_ at 392 and B1_g2_ at 511 cm^−1^ are related to the O–Ti–O symmetric bending vibration, and the one A1g mode at 512 cm^−1^ is related to the O–Ti–O anti-symmetric bending vibration^37,38^. In the case of Co-doped TiO_2_ sample, it is found that the main peaks E_g1_ and E_g2_, located at 144 cm^−1^ and 196 cm^−1^, are shifted to higher wavenumbers (147.2 and 199.2 cm^−1^). However, the E_g3_ peak (632 cm^−1^) is shifted to a lower wavenumber (629.8 cm^−1^) compared to that of bare TiO_2_. Besides, the B_1g_ and A_1g_ peaks are shifted to 389.6 and 509.7 cm^−1^, respectively. Furthermore, no bands appeared related to any cobalt oxide phase, probably due to the low Co content in TiO_2_ lattice^39^. As the ionic radius of Co^2+^ (0.70 Å) is larger than that of Ti^4+^ (0.64 Å), the insertion of Co as a dopant should lead to a structural distortion and induce oxygen vacancies, which can be the main reason of the observed peak shift. Moreover, electron–phonon coupling is one of the physical parameters used to understand the electron transport and the existence of oxygen vacancies in the lattice of metal oxides^20^. The electron–phonon coupling is related to the phonon linewidth (FWHM) and can be estimated from the energy-time uncertainty relation (Eq. 6) ^40^:

6 $$ {1}/ \, \tau \, = { 2}\pi {\text{cr,}} $$

where *τ* is the phonon lifetime, *c* is the speed of light (3 × 10^8^ m/s), and *r* is the FWHM of the Raman peak in units of cm^−1^. The estimated phonon lifetime is found to decrease from 2.49 to 2.11 ps upon cobalt doping.

The FT-IR spectra of the bare and Co-doped TiO_2_ mesocrystals are shown in Fig. 1c. The broad peaks located at 3847, 3837, and 3800 cm^−1^ are ascribed to Ti^3+^^41^. Besides, the broad peaks at 3435 and 3750 cm^−1^ are likely due to stretching vibrations of adsorbed O–H groups, while the peak at 1748 cm^−1^ arises from Ti–O–C vibration, confirming the effective interaction between Ti and C. The peak at 1643 cm^−1^ is from the H–O–H bending mode^42^. The peak located at 1427 cm^−1^ is assigned to the Ti–O vibrations on the {001} facets^43^, in good agreement with the XRD results. Also, the peak at 1024 cm^−1^ is due to Ti–F vibrations in both samples. Moreover, the Ti–O stretching and Ti–O–Ti bridging have appeared between 521 and 460 cm^−1^. Thus, the FT-IR spectra reveals the presence of Ti^3+^ in the prepared TiO_2_ mesocrystals.

**Figure 2:**
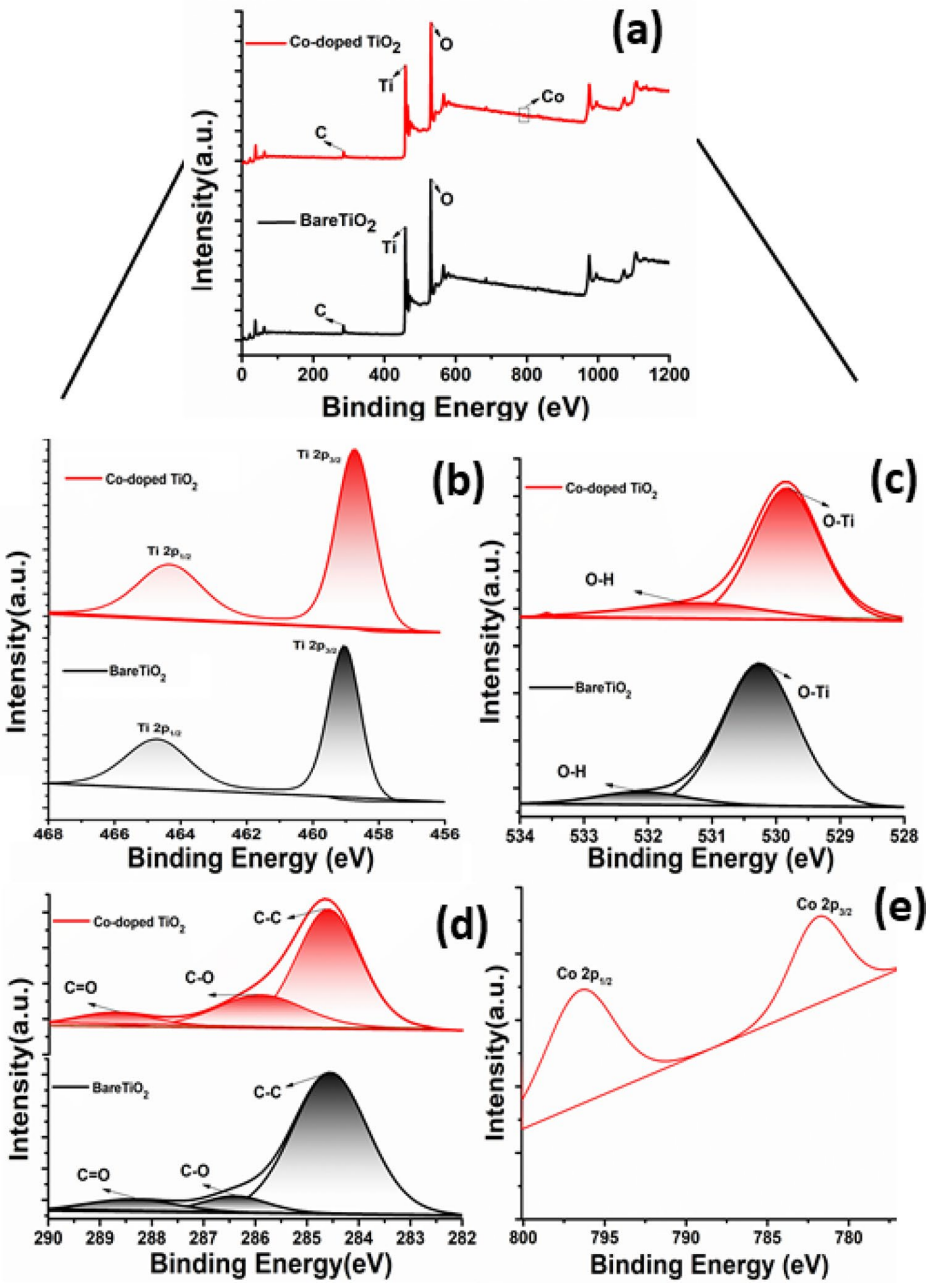
XPS spectra of bare and Co-doped TiO_2_mesocrystals: (**a**) survey spectra, and the HR-spectra of (**b**) Ti 2p, (**c**) O 1 s, (**d**) C 1 s, and (**e**) Co 2p.

In order to investigate the bonding states of the elements on the surface of bare and Co- doped TiO_2_, XPS analysis was carried and the data are presented in Fig. 2. The full-scan XPS survey of both samples (Fig. 2a) revealed peaks that are exclusively related to Ti, O, and C, elements in addition to an extra peak of Co in the Co- doped TiO_2_ sample. Figure 2b shows the high-resolution XPS spectra of Ti 2p, where Ti 2p_3/2_ and Ti 2p_3/2_ peaks are observed at 459.1 eV and 464.7 eV, respectively for the bare TiO_2_ sample. The O1s HR-XPS spectrum (Fig. 2c) is fitted into two-sub peaks centered at 530.26 eV and 532.17 eV for bare TiO_2_, and 529.78 and 531.4 eV for Co-doped TiO_2_. The peaks at 530.26 eV and 529.78 eV are related to Ti–O and surface OH^-^groups^44^. Additionally, the other oxygen peak (530.3 eV) in Co-doped TiO_2_ is originated from the presence of Co–O bond^45^. Moreover, the C 1 s peak (Fig. 2d) can be de-convoluted into three peaks in both samples, one located at 284.69 eV and 284.54 eV and others at 285.82 eV, 286.39 eV, 288.67 eV and 288.42 eV for bare and Co-doped TiO_2_, respectively. The main peak corresponds to C–C bond that exists in carbon species and the others at higher energy could arise from C–O and C=O bond in TiO_2_, revealing interstitial and/or substitutional C ^46^. Finally, the Co 2p peaks appeared at 781.8 and 796.42 eV (Fig. 2e) correspond to Co^2+^ 2p_3/2_ and Co^2+^ 2p_1/2_, respectively. Notably, all elements binding energies (Ti, O, and C) in Co-doped TiO_2_ sample exhibited a slight negative shift when compared to the bare TiO_2_ sample, which can be related to the doping of cobalt ions in TiO_2_ lattice where the Co has a higher electronegativity than Ti^47^.

**Figure 3:**
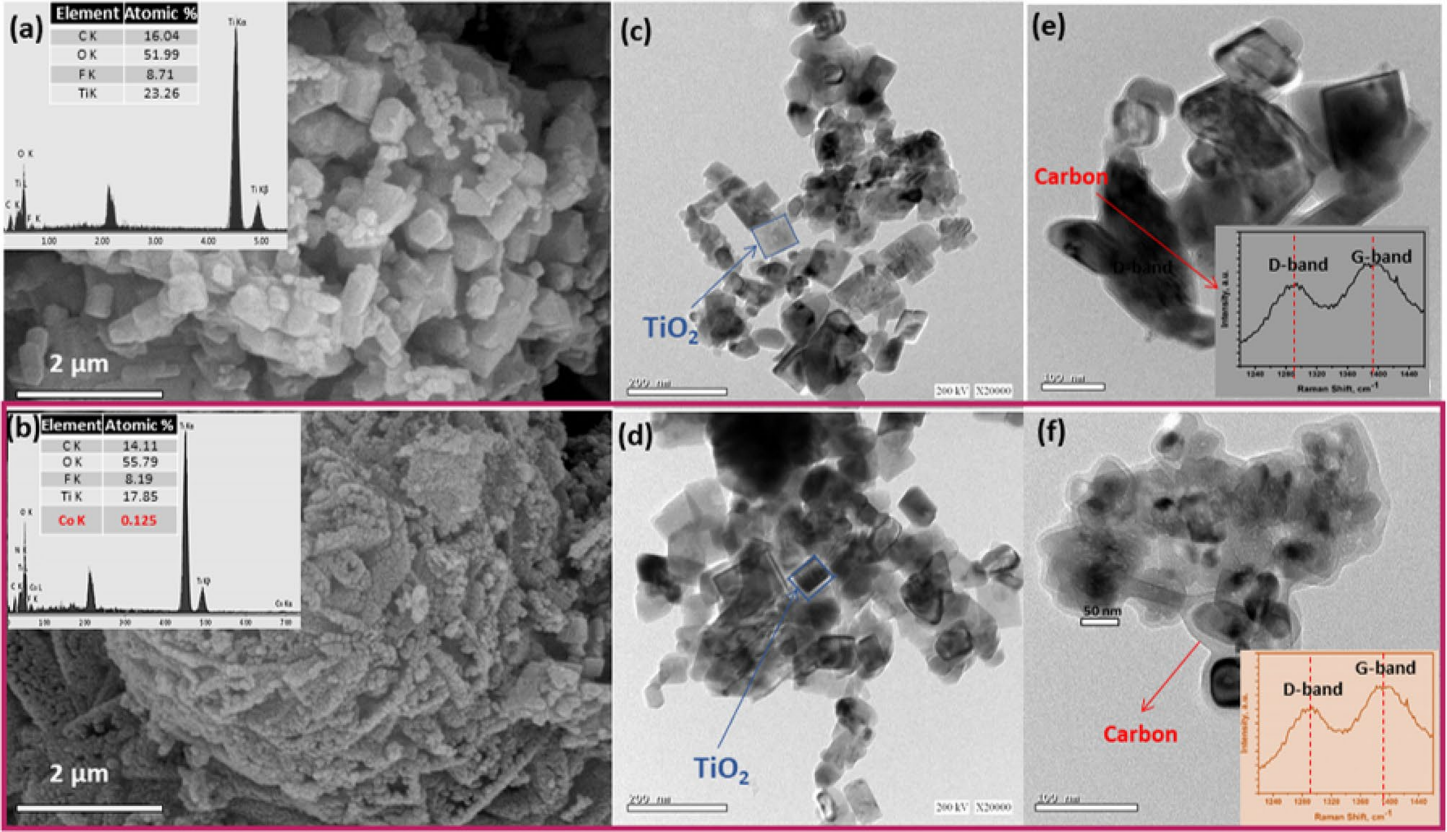
(**a**,**b**) FESEM images, (**a**,**b** insets) EDX analysis, (**c**–**f**) HR-TEM images and (**e**,**f** insets) Raman spectra of bare and Co-doped TiO_2_ mesocrystals.

Figure 3a,b shows the representative FESEM images of the prepared undoped and cobalt-doped TiO_2_ mesocrystals, respectively, revealing agglomerates of highly connected of small particles with homogenous size distribution. Upon Co-doping, a notable decrease in the size of the particles was observed, revealing the effect of cobalt insertion on retarding the TiO_2_ growth^48^. The EDX analysis (insets in Fig. 3e,f) reveals the presence of Ti, O, C, and Co without any impurities. Figure 3c–f depicts typical HR-TEM images of the TiO_2_ mesocrystals before and after doping with cobalt, viewed along a square surface of {001} crystallographic direction. Note also the presence of a carbon shell as indicated by an arrow in Fig. 3e,f. The transparent carbon layer is uniform and continuously surrounding the TiO_2_ mesocrystals^49,50^. Raman spectroscopy was used to confirm the presence of the residual carbon (insets in Fig. 3e,f), where the peaks at 1400 and 1290 cm^−1^ are mainly originating from sp^3^ hybridization (D-band) and the planar configuration of the sp^2^-bonded carbon structure (G-band), respectively^51^.

The optical absorption of the bare and Co-doped TiO_2_ mesocrystals was elucidated by recording their UV–Vis diffuse reflectance spectra (DRS) as shown in Supplementary Fig. S1a. Both samples exhibit visible light response in the wavelength range of 400–600 nm. The spectra of bare TiO_2_ sample (Supplementary Fig. S1a) reveal an absorption edge at 418 nm, which is redshifted relative to that reported for P25 (TiO_2_)^52^. The Co-doped TiO_2_ sample showed an absorption edge at 424 nm, which is redshifted relative to the bare TiO_2_. The UV–visible DRS analysis illustrated that bare and Co-doped TiO_2_ have bandgaps of 2.75 and 2.6 eV, respectively (Supplementary Fig. S1b). The observed redshift in the absorption spectra can be ascribed to Co^2+^  → Ti^4+^ charge-transfer^53^. Thus, photoluminescence (PL) emission spectroscopy was utilized to study the fate of the photoinduced charge carriers^54^. Oxygen vacancies and surface states play a vital role in the photocatalytic response of anatase TiO_2_^55^. Supplementary Fig. S1c shows the room-temperature PL spectra of the bare and Co-doped TiO_2_ samples, where eight peaks started from 396 nm and ended at 700 nm were recorded^56^. The detected superimposed multi-peaks may reveal radiative recombination of electron–hole pairs from different energy levels^57^. Notably, the Co-doped TiO_2_ exhibited a decrease in the PL peaks intensity compared to the bare TiO_2_, indicating a reduction in the recombination rate of charge carriers in the Co-doped TiO_2_ sample^58^. These results suggest the superior photocatalytic activity of Co-doped TiO_2_ mesocrystals over bare TiO_2_ mesocrystals.

### Antimicrobial properties

**Figure 4:**
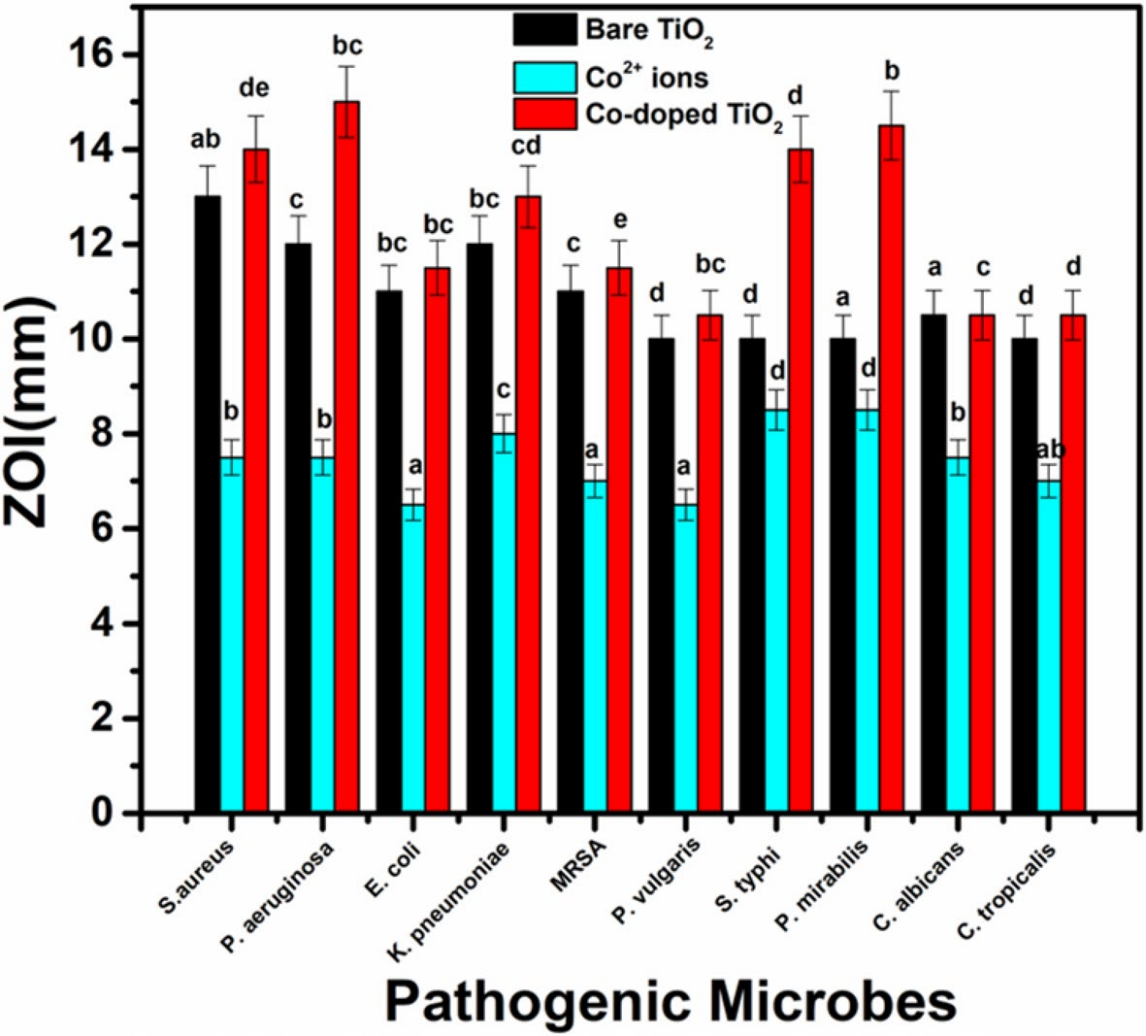
Antimicrobial activity of bare TiO_2_ NPs and Co-doped TiO_2_ NPs against different pathogenic microbes as ZIO. The data within the groups are analyzed using a one-way analysis of variance (ANOVA) followed by^a,b,c,d,e^ Duncan’s multiple range test.

Usually antimicrobial agents are used to hinder microbial diseases emanating from clinical poisoning, such as urinary tract infection (UTI)-causing microbes^31^. However, nanomaterials-based agents have recently received great attention as they are uniquely-applied to combat pathogenic microbes^59^. In our study, the fabricated samples were checked for their antimicrobial capabilities using the disc agar diffusion technique. The TiO_2_ samples were found to deactivate a broad spectrum of the tested bacteria such as *P. aeruginosa*, *P. mirabilis*, and *S. aureus*. Specifically, Co-doped TiO_2_ mesocrsytals showed the most powerful antibacterial effect against all examined microbes, see Fig. 4, Supplementary Fig. S2 and Supplementary Table S1. The antimicrobial abilities of the samples were compared with Co^2+^ ions and standard antibacterial and antifungal agents like Amoxicillin (AX; 25 μg/mL) and Nystatin (NS; 25 μg/mL). Our samples are found to be more active than the used standard antibiotics, and Co^2+^ ions. Interestingly, the synthesized TiO_2_ mesocrystals were found to be more active against Gram-negative bacteria than the Gram-positive counterpart because the cell wall of the Gram-negative bacteria contains a thick layer of lipopolysaccharide essentially in addition to a small layer of peptidoglycan. On contrary, Gram-positive bacteria primarily incorporate a thicker layer of peptidoglycan blocks^29^. The fabricated NPs enjoy high surface-to-volume ratio, thus can be easily combined and interact with some of the pathogenic microbes, such as yeasts, bacteria, and fungi yeast^30^.

The MIC results ranged from 0.39 to 25 μg/mL of the integrated samples against all tested microbes. The promising MIC of the Co-doped TiO_2_ mesocrystals was 0.39 μg/mL (*P. aeruginosa),* and 0.781 μg/mL (*P. mirabilis).* Additionally, the synthesized Co-doped TiO_2_ NPs exhibit accepted MIC of 12.5 μg/mL against *C. albicans* at very low NPs concentration (10.0 µg/mL)*,* suggesting their potential use as antifungal agents. Importantly, the properties of the synthesized mesocrystals play a vital role in their antimicrobial characteristics, including their structure, purity, and size^51–54^. Various advanced mechanisms, such as reactive oxygen species (ROS) distribution (superoxide anion; O_2_^−^), were proposed in the literature to explain the possible effects of a plethora of metal oxides as antibacterial agents^60–63^. However, the antimicrobial mechanism of Co-doped TiO_2_ mesocrystals has not been identified yet. Thus, the interaction of Co-doped TiO_2_ mesocrystals with the pathogenic microbes and the alkaline tendency have been included here to demonstrate the possible antimicrobial activity mechanism. It is suggested that Co-doped TiO_2_ mesocrystals could alter the microbial morphology and their film composition, change the microbial membrane permeability, and produce the residence of oxidative stress genes via the production of H_2_O_2_^60^. Note that Co^2+^ ions were shown to possess antibacterial activity, where a series of Co^2+^ complexes of mercapto-thiadiazole-derived furanyl, thienyl, pyrrolyl, salicylic, and pyridinyl Schiff bases exhibited in-vitro weak to moderate antibacterial potential toward Gram-negative bacteria (*Escherichia coli*, *Pseudomonas aeruginosa*, *Salmonella typhi*, and *Shigella flexneri*) and Gram-positive bacteria (*Bacillus subtilis* and *Staphylococcus aureous*)^64^. Another study by Gaëlle et al.^65^ showed that the ligands, metal salt, and the complexes (Cobalt (II) complex [Co(phen)_3_(NO_3_)_2_]·2H_2_ O and a novel Co (III) complex) were investigated for their antimicrobial potentials in-vitro toward pathogenic bacteria and fungi. The antimicrobial results indicated that all ligands were very effective towards the tested microbes. The antibacterial activity of pure cobalt or Co^2+^ ions was attributed to the reaction with negatively-charged molecules inside the microbial cells, which in turn leads to genotoxicity and destruction of the main bacterial organelles^31^.

### Antibiofilm activity of Co-doped TiO_2_ mesocrystals

**Figure 5:**
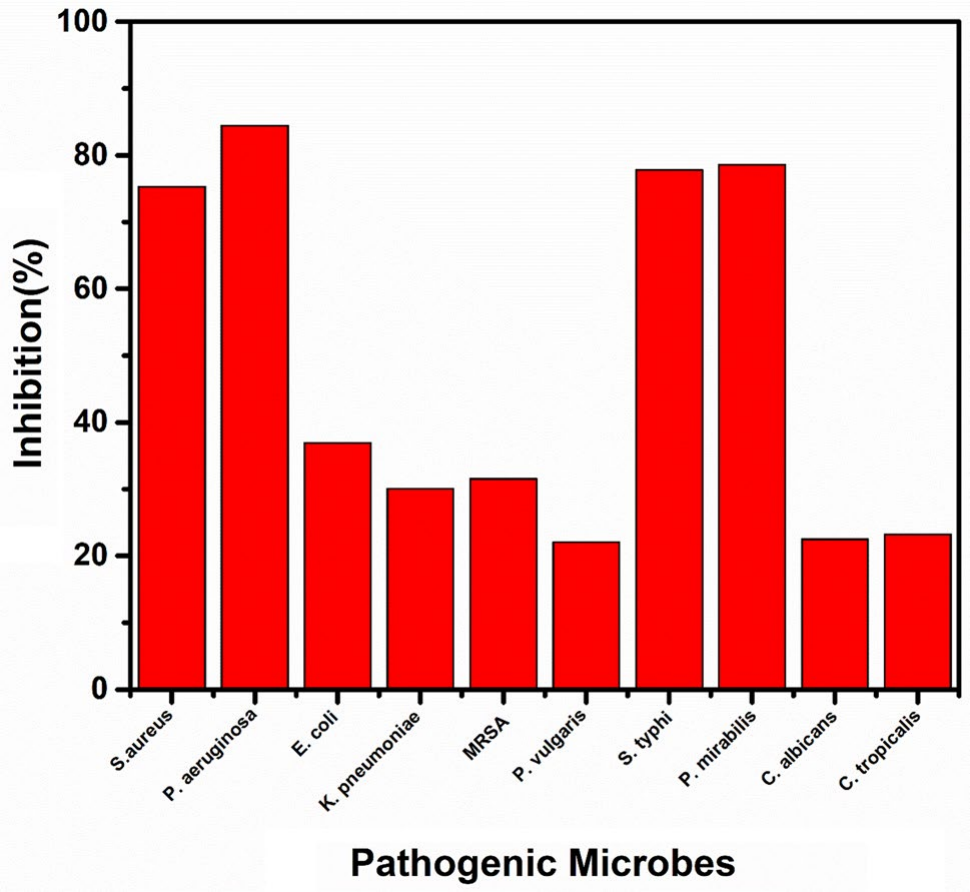
Antibiofilm activity of Co-doped TiO_2_ against different pathogenic microbes as inhibition %.

The formation of biofilm in pathogenic microbes is characterized by the exo-polysaccharide secretion^28,29^. The tube method was applied to determine the antibiofilm potential of the synthesized Co-doped TiO_2_ mesocrystals against some UTI-producing microbes. Supplementary Fig. S3 shows the antibiofilm action of the Co-doped TiO_2_ mesocrystals for *Pseudomonas aeruginosa* and *Candida albicans.* The complete steps are: (I) Normal microbial growth and production of the distinct ring in the lack of the synthesized Co-doped TiO_2_ mesocrystals and the interference with microbial growth in the closeness of Co-doped TiO_2_ mesocrystals, (II) The probability of staining of the formed biofilm with Crystal Violet (CV), which is a qualitative determination method, and (III) Eliminating and separating the adhered microbial cells after ethanol addition for semi-quantitative estimation of the biofilm hindrance % (Supplementary Table S2). Supplementary Fig. S3a shows the tube design for the determination of antibiofilm potential of Co-doped TiO_2_ against *P. aeruginosa,* the sensitive bacteria example, which creates a thick whitish-yellow layer in the air–liquid interface in the lake of the mesocrystals (control). The produced matt layers were fully-adhered across the walls of the designed tubes and developed as a blue color following the staining with CV. Next, a dark blue color was created in the produced solution subsequent dissolving CV with absolute ethanol, as presented in Supplementary Fig. S3a. On the other side, in the tubes including *P. aeruginosa* cells and in the closeness of Co-doped TiO_2_ mesocrystals (10 µg/mL), a remarkable negative effect was recognized as the cells of the tested bacteria do not form biofilm layers and the ring formation was blocked. Also, the adherent cell color was quiet and the blue color was faintly-formed after ethanol addition, as displayed in Supplementary Fig. S3a. Related forms were shown for the biofilm repression of the tested yeast *C. albicans* as presented in Supplementary Fig. S3b. The semi-quantitative determination of the inhibition percentage (%) was investigated by a UV–visible spectrophotometer. The optical density (O.D.) was estimated at 570 nm following terminating CV-stained biofilms, which were considered as a means of their creation. Supplementary Table S2 displays the inhibition% following the addition of 10.0 µg/mL Co-doped TiO_2_ mesocrystals, revealing that the highest percentage for *P. aeruginosa* is 84.43%, for *P. mirabilis* is 78.58%, and for *S. typhi* is 77.81%. Note that Co-doped TiO_2_ mesocrystals were able to control the biofilm growth at its adhesion degree, which is the first step in the antimicrobial process^66^. The change in the inhibition percentage may be ascribed to many factors such as the high potential of the antimicrobial agents to be attached to the surface due to the high surface area of the synthesized Co-doped TiO_2_ mesocrystals and their particle size as well as the invasion skills and different chemical characteristics influencing the relationship and communication of Co-doped TiO_2_ mesocrystals with biofilms-producing microbes^67^. Positively, the synthesized Co-doped TiO_2_ mesocrystals suppressed the growth of *P. aeruginosa* by more than 98% with 0.39 µg/mL MIC as listed in Supplementary Table S2. Figure 5 shows a summarized diagram regarding the antibiofilm potential of Co-doped TiO_2_ mesocrystals (as inhibition %) against different pathogenic microbes.

### Antimicrobial effect of Co-doped TiO_2_ in liquid media under illumination

**Figure 6:**
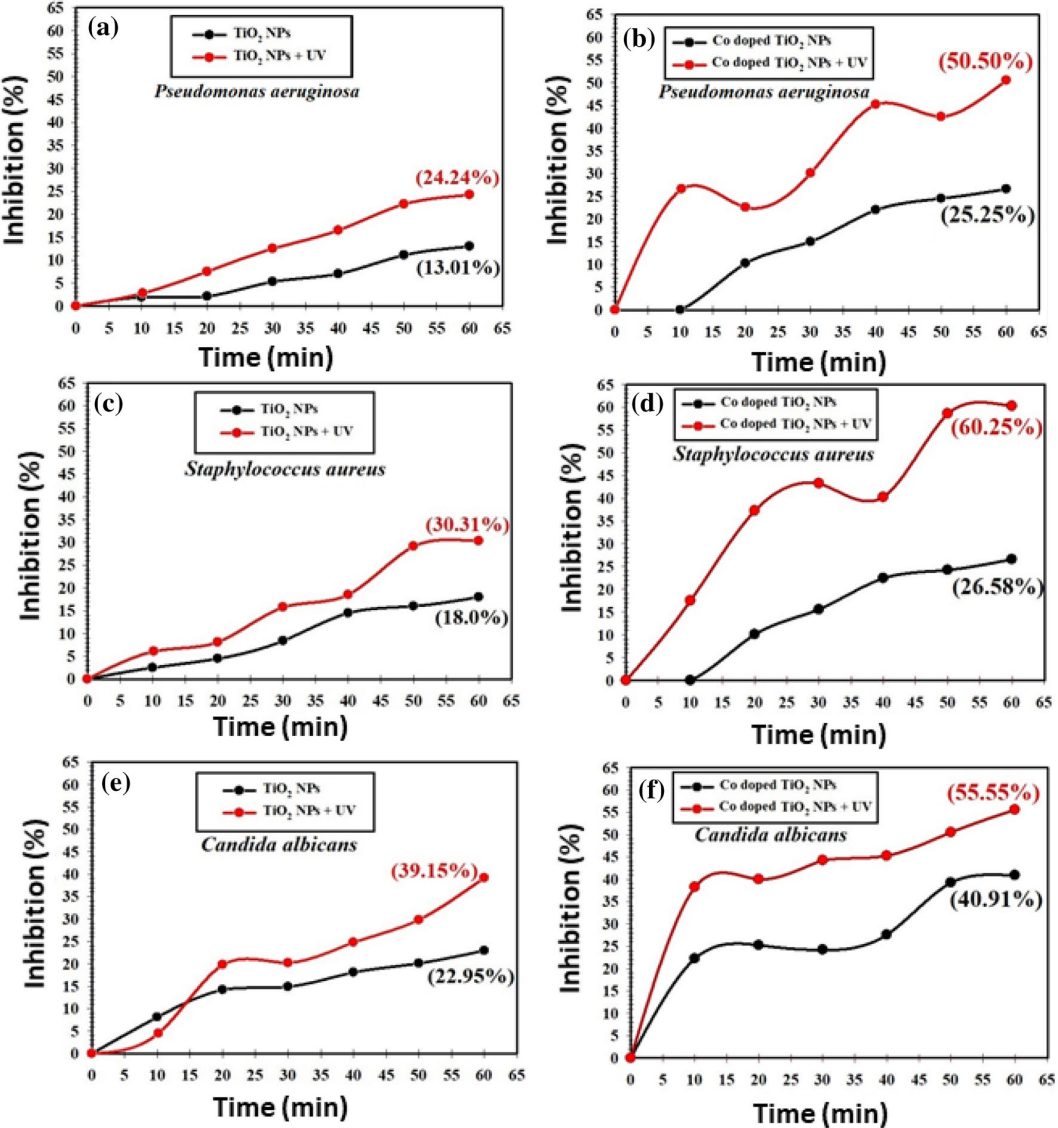
Antimicrobial effect under UV-irradiation effect against different pathogenic microbes, where *Pseudomonas aeruginosa* (**a**,**b**), *Staphylococcus aureus* (**c**,**d**), and *Candi1da albicans* (**e**,**f**), using of bare TiO_2_, and Co-doped TiO_2_ in liquid media, respectively.

The comparison between the inhibition% of *P. aeruginosa*, *S. aureus*, and *C. albicans* upon the use of TiO_2_ and Co-doped TiO_2_ mesocrystals and UV are presented in Fig. 6. Note that Co-doped TiO_2_ showed higher antimicrobial activities against *P. aeruginosa, S. aureus,* and *C. albicans* colonies than pure TiO_2_, Fig. 6b–d, revealing the synergistic actions of Co doping and the TiO_2_ mesocrystals. Moreover, upon UV-illumination, Co-doped TiO_2_ mesocrystals exhibited even higher antimicrobial activities than that in the dark. The maximum inhibition percentage of bare TiO_2_ and Co-doped TiO_2_ mesocrystals under UV-illumination for *P. aeruginosa* at the end of the experiment was 24.24% and 50.50%, respectively (Fig. 6a,b), while it was 30.31% and 60.25% for *S. aureus* (Fig. 6c,d), and 39.15% and 55.55% for *C. albicans* (Fig. 6e,f).

The observed activity under light irradiation may be related to the induced oxygen species such as OH free radicals, which caused the destruction of the microbial coenzymes and reduced their contents^19,42^. The major influences involve the creation of holes in the cell wall of the microbes, which subsequently-progressed the cell permeability and finally a cell death will occur. To confirm the induced oxygen species, electron paramagnetic resonance (EPR) spectra were collected as shown in Supplementary Fig. S4. The two samples showed signals at g = 1.95, g = 2.157, g = 2.05, and g = 2.12, confirming the presence of Ti^3+^ and free oxygen radicals such as OH^**.**^, O^**.-**^, or O_2_^.–^^68^. Notably, the Co-doped TiO_2_ sample showed a higher concentration of paramagnetic centers of 4.16953 × 10^18^ spin/g relative to the bare TiO_2_ sample (4.09473 × 10^18^ spin/g). The increased paramagnetic centers after the addition of cobalt may be related to the increase in the concentration of oxygen radicals in the TiO_6_ lattice^57^. This is in agreement with the inhabitation % of Co-doped versus bare TiO_2._

**Figure 7:**
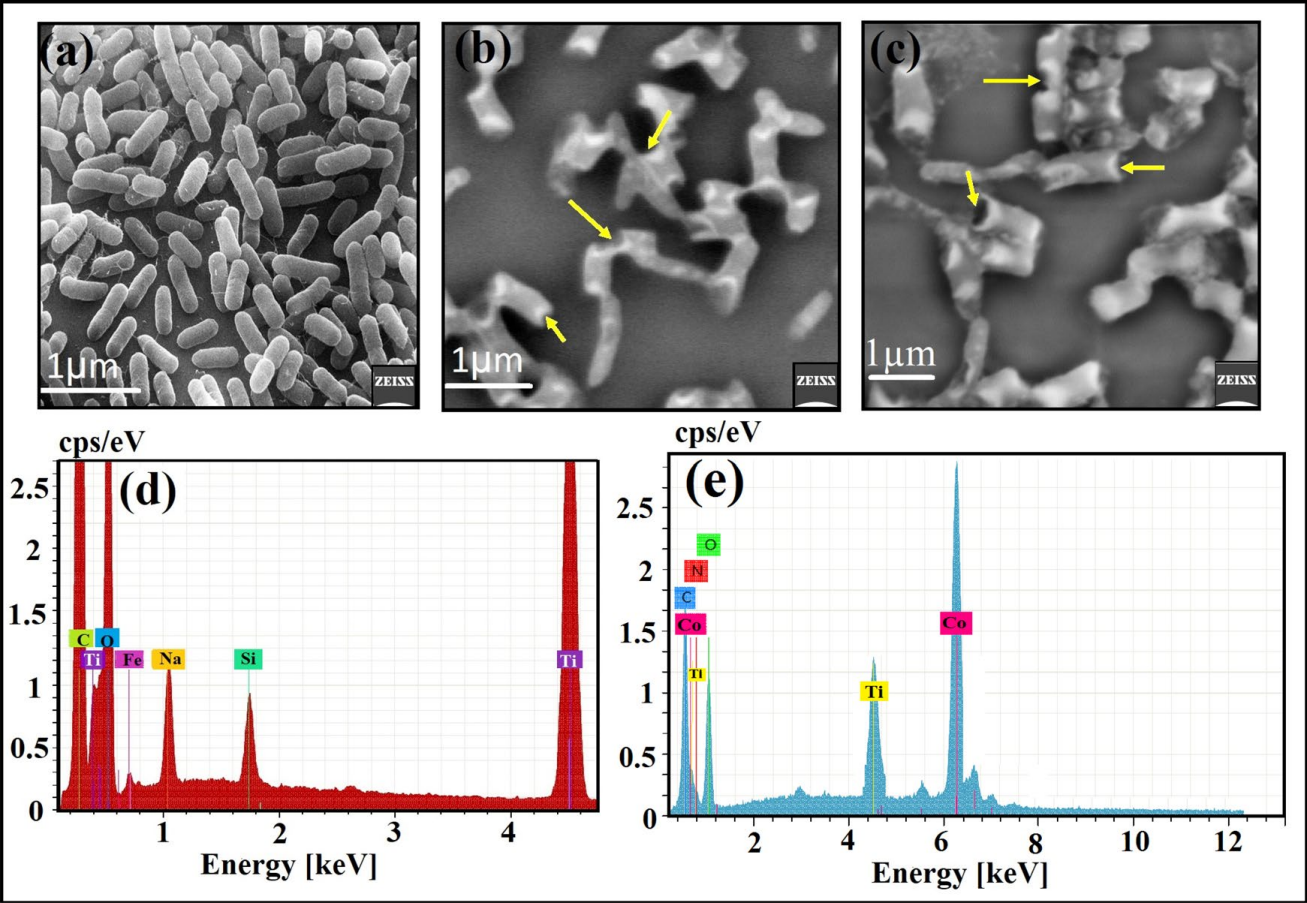
SEM and the matching EDX elemental study of *P. aeuroginosa*: (**a**) Regular bacterial cells (*P. aeuroginosa*) without TiO_2_ mesocrystals, and Co-doped TiO_2_ mesocrystals treatment, (**b**) Abnormal, deformed and irregular bacterial cell with incomplete lysis following TiO_2_ mesocrystals treatment, (**c**) Fully-irregular and deformed bacterial cell through Co-doped TiO_2_ mesocrystals treatment presenting the full lysis of *P. aeuroginosa* cell, (**d**) Matching EDX elemental study of the treated *P. aeuroginosa* cell validating the cellular internalization of the qualified TiO_2_ mesocrystals in *P. aeuroginosa* cells, and (**e**) Matching EDX elemental examination of the treated *P. aeuroginosa* cell establishing the cellular internalization of the integrated Co-doped TiO_2_ mesocrystals in *P. aeuroginosa* cells.

SEM/EDX analysis was performed to elucidate the possible antimicrobial mechanism toward *P. aeuroginosa*, see Fig. 7. The SEM analysis of the control sample in the absence of any mesocrystals showed bacterial groups that are constantly-developed with typical normal bacterial surface and semi-formed biofilm, Fig. 7a. Upon TiO_2_ mesocrystals treatment, noticeable morphological differences were identified in *P. aeuroginosa* (Fig. 7b), including the incomplete lysis of the outer surface followed by deformations of the *P. aeuroginosa* cells*.* Additionally, Co-doped TiO_2_ mesocrystals caused the entire and complete lysis of the bacterial cell with the decrease in the whole viable number, and ultimately the biofilm growth was restrained (Fig. 7c). The EDX elemental analysis shows the presence of Ti and O elements with others from the bacteria like C, and O, along with Fe, Si, and Na form the microelement in the bacterial medium. All detected elements were located at the malformed cities and the outside surface of the *P. aeuroginosa* cells, validating the performance of the tested TiO_2_ mesocrystals, (Fig. 7d). Finally, the EDX elemental spectra, in case of Co-doped TiO_2_, revealed Co, Ti, and O elements along with different atoms from the bacterial structure at the irregular areas and at the outside surface of the treated *P. aeuroginosa* cells.

**Figure 8:**
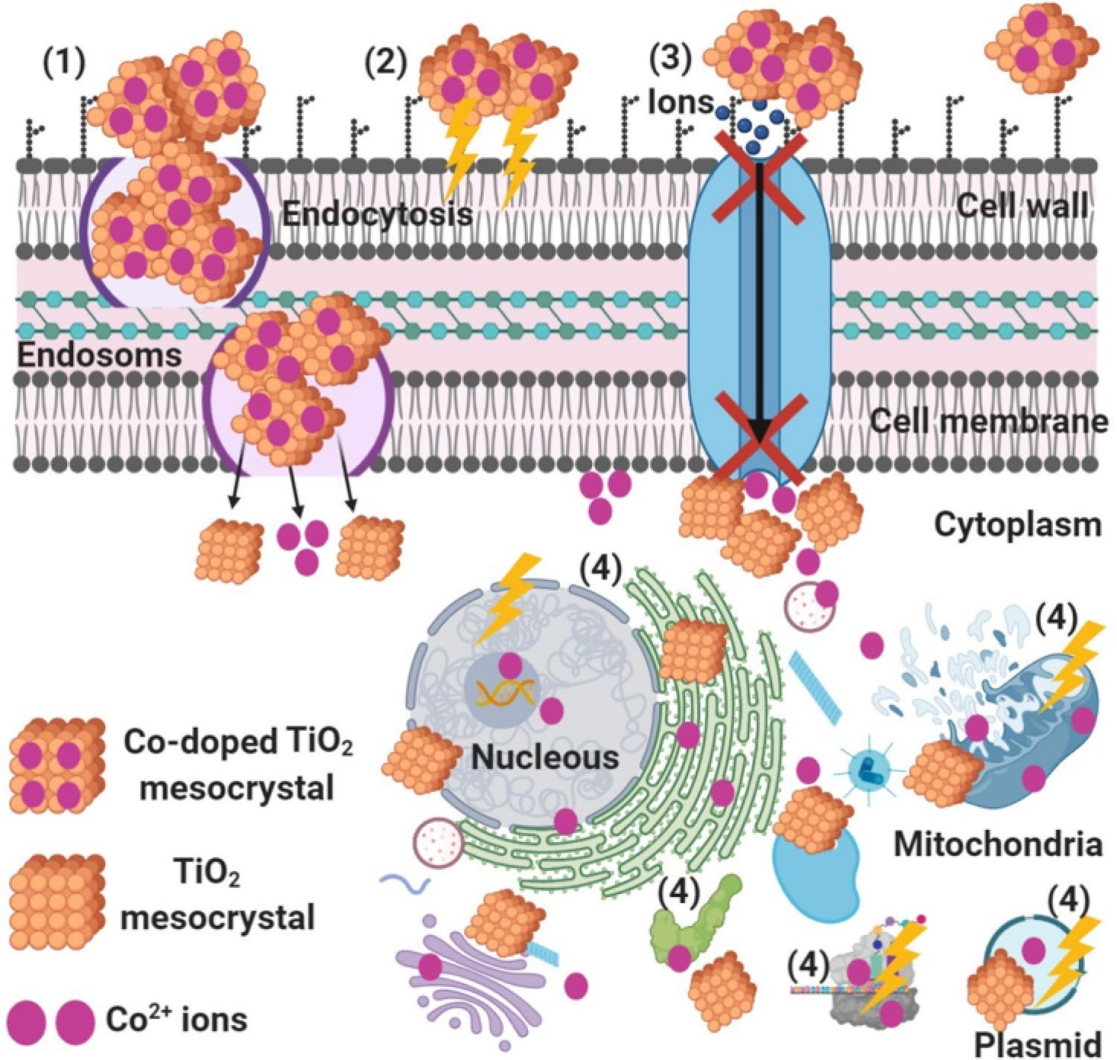
Schematic representation regarding the four prominent ways of antimicrobial potential of Co-doped TiO_2_ mesocrystals, where (1) Co-doped TiO_2_ mesocrystals adhere to the bacterial cell surface and results in membrane damage and altered transport activity; (2) Co-doped TiO_2_ mesocrystals create and increase the ROS leading to cell damage, (3) Co-doped TiO_2_ mesocrystals block the ions transport from and to the bacterial cell, and (4) Co-doped TiO_2_ mesocrystals penetrate inside the bacterial cells and interact with cellular organelles and biomolecules, and thereby, affect respective cellular machinery, and modulate the cellular signal system and causing cell death. Co-doped TiO_2_ mesocrystals may serve as a vehicle to effectively-deliver Co ions to the bacterial cytoplasm and membrane, where proton motive force would decrease the pH to be less than 3.0 and therefore improve the release of Co ions.

The schematic in Fig. 8 illustrates the possible antibacterial mechanism. We believe that Co-doped TiO_2_ mesocrystals start their action by adhesion at the outer surface of the bacterial cell, causing membrane damage and altered transport activity. Then, diffusion of Co^2+^ inside the bacterial cell (at pH = 3) and dividing all of the intracellular structure like mitochondria, plasmid, DNA, and other vital organelles. Afterwards, cellular toxicity occurs due to the oxidative stress generated by the production of ROS. Finally, TiO_2_ mesocrystals were withstood the acidic condition inside the bacterial cells and conversion did not occur^69^ but possessed the antibacterial effect by affecting the signal transduction pathways.

## Conclusion

In summary, unique multi-doped TiO_2_ mesocrystals have been synthesized via a facile sol–gel approach. The crystal structure, optical, and compositional properties of the materials were elucidated using XRD, Raman, FTIR, XPS UV–vis analyses. The synthesized Co-doped TiO_2_ mesocrystals showed excellent antimicrobial activity compared to bare TiO_2_ counterparts. The antimicrobial performance was evaluated in terms of zone of inhibition, minimum inhibitory concentration (MIC), antibiofilm activity, and photoactivity. Co-doped TiO_2_ mesocrystals showed 60.25% inactivation of *S. aureus* after 60 min of UV illumination. The SEM findings supported the results of the viability tests, demonstrating complete lysis of the bacterial cells with the decrease in the whole viable number. The Co-doped TiO_2_ mesocrystals showed very promising MIC of 0.390 μg/mL and 0.781 μg/mL for *P. mirabilis* and *P. mirabilis*, respectively*.* Additionally, the material showed an MIC of 12.5 μg/mL against *C. albicans*, suggesting its use as antifungal agent. Considering the efficient fast and oxidative damage mediated inactivation of bacteria on the Co-doped TiO_2_ mesocrystals showed in this study, our results supports further development and application of Co-doped TiO_2_ mesocrystals in other fields.

## Supplementary Information

Supplementary Information.
