# Supplementary material for: Superior visible light antimicrobial performance of facet engineered cobalt doped TiO2 mesocrystals in pathogenic bacterium and fungi
Source: Sci Rep. 2021 Mar 10;11:5609. doi: 10.1038/s41598-021-84989-x (PMC7946932; doi:10.1038/s41598-021-84989-x)
Supplement: Supplementary file 1 — Supplementary Information. [file 41598_2021_84989_MOESM1_ESM.docx]

**Supplementary material**

**Superior visible light antimicrobial performance of facet engineered cobalt doped TiO_2_ mesocrystals in pathogenic bacterium and fungi**

Ayat N. El-Shazly ^1, 2†^, Gharieb S. El-Sayyad ^3†^, Aiat H. Hegazy ^4†^, Mahmoud A. Hamza ^5^, Rasha M. Fathy ^3^, E. T. El Shenawy ^4^, Nageh K. Allam ^1*^

^1^ Egypt Energy Materials Laboratory, School of Sciences and Engineering, The American University in Cairo, New Cairo 11835, Egypt

^2^ Central Metallurgical Research and Development Institute, P.O. Box 87, Helwan, Cairo, Egypt

^3^Drug Microbiology Lab., Drug Radiation Research Department, National Center for Radiation Research and Technology (NCRRT), Egyptian Atomic Energy Authority (EAEA), Cairo, Egypt

^4^ Solar Energy Department, National Research Centre, Giza, Dokki, Egypt

^5^ Chemistry Department, Faculty of Science, Ain Shams University, Abbasia, Cairo, Egypt

*Corresponding Author E-mail: [nageh.allam@aucegypt.edu](mailto:nageh.allam@aucegypt.edu) (N. K. Allam)


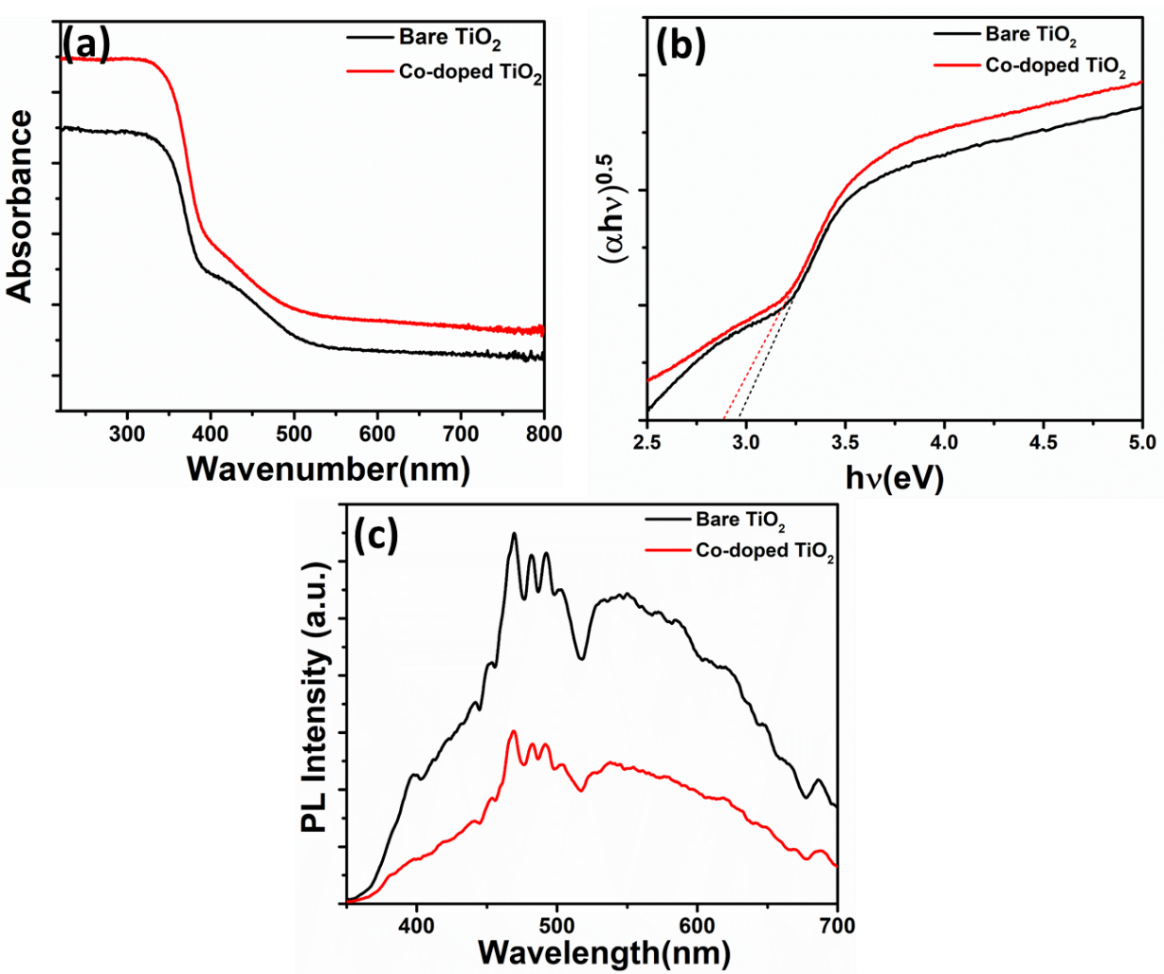


**Figure S1.** (a) Absorbance spectra, (b) Tauc plots, and (c) PL spectra of the bare and Co-doped TiO_2_ mesocrystals

**
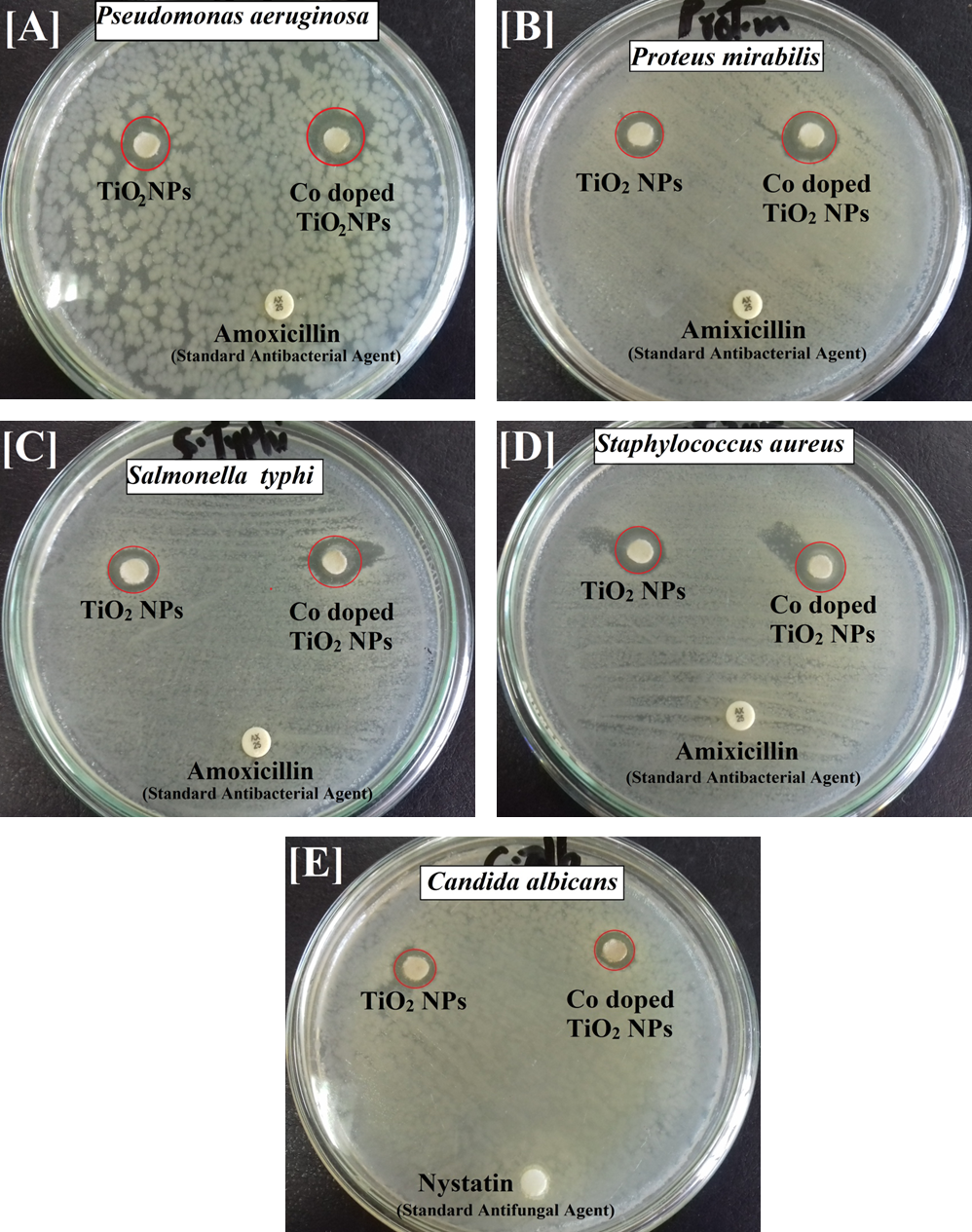
**

**Figure S2.** Antimicrobial activities, ZOI (mm), of TiO_2_ and Co-doped TiO_2_ mesocrystals against some pathogenic microbes: (a) *Pseudomonas aeruginosa*, (b) *Proteus mirabilis*, (c) *Salmonella typhi*, (d) *Staphylococcus aureus*, and (e) *Candida albicans*.


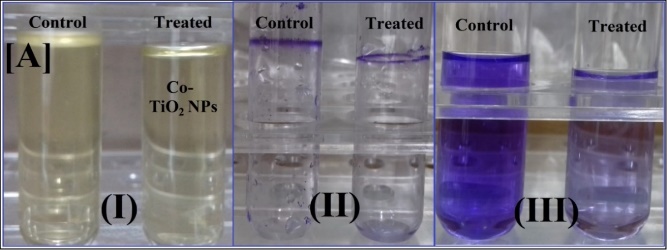

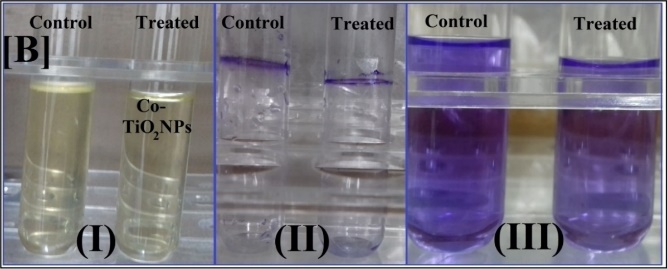


**Figure S3.** Antibiofilm potential of the synthesized Co-doped TiO_2_ mesocrystals using tube method against (a) *Pseudomonas aeruginosa* (example of bacteria) and (b) *Candida albicans* (example of yeast).





**Figure S4.** EPR spectrum of the bare TiO_2_ and Co-doped TiO_2_ mesocrystals

**Table S1.** Antibacterial and antifungal activities of bare, Co-doped TiO_2_, and Co^2+^ ions against some multi-drug resistant (MDR) bacteria and pathogenic *Candida* species as ZOI (mm) and MIC (μg/ml).

| **Pathogenic microbes** | **ZOI (mm), [TiO_2_]= 10µg/ml** | | **MIC** of Co-doped TiO_2_ (100 µg/ml) | **ZOI (mm)** of Co^2+^ ions  (10µg/ml) | **ZOI (MM)** of AX & NS | **MIC** of AX & NS  (100 µg/ml) |
| --- | --- | --- | --- | --- | --- | --- |
|  | bare TiO_2_ | Co-doped TiO_2_ |  |  |  |  |
| *Staphylococcus aureus* | 13.0^ab^ ± 0.5773 | 14.0^de^ ± 0.5773 | 1.562 | 7.5^b^ ± 0.5000 | Nil | 75.0 |
| *Pseudomonas aeruginosa* | 12.0^c^ ± 0.5773 | 15.0^bc^ ± 0.5773 | 0.390 | 7.5^b^ ± 1.1547 | Nil | 50.0 |
| *Escherichia coli* | 11.0^bc^ ± 0.5773 | 11.5^bc^ ± 0.2886 | 6.250 | 6.5^a^ ± 0.6110 | Nil | 50.0 |
| *Klebsiella pneumoniae* | 12.0^bc^ ± 0.5773 | 13.0^cd^ ± 0.7637 | 3.125 | 8.0^c^ ± 0.6110 | Nil | 75.0 |
| *Staphylococcus aureus* (MRSA) | 11.0^c^ ± 0.5773 | 11.5^e^ ± 1.1547 | 6.250 | 7.0^a^ ± 0.5000 | Nil | 50.0 |
| *Proteus vulgaris* | 10.0^d^ ± 0.5773 | 10.5^bc^ ± 0.5000 | 12.50 | 6.5^a^ ± 0.5000 | Nil | 100 |
| *Salmonella typhi* | 10.0^d^ ± 0.5773 | 14.0^d^ ± 0.5773 | 1.562 | 8.5^d^ ± 0.5773 | Nil | 50.0 |
| *Proteus mirabilis* | 10.0^a^± 0.5773 | 14.5^b^ ± 0.6110 | 0.781 | 8.5^d^ ± 0.4509 | Nil | 50.0 |
| *Candida albicans* | 10.5^a^± 0.5773 | 10.5^a^ ± 0.4509 | 12.50 | 7.5^b^ ±0.5773 | Nil | 100 |
| *Candida tropicalic* | 10.0^d^ ± 0.5773 | 10.5^d^ ± 0.5773 | 25.00 | 7.0^ab^ ±0.2886 | Nil | 75.0 |
| LSD | 1.00000 | 1.33333 | ----------- | 1.66636 | ------- | -------- |

*Values are means ± SD (n = 3). Data within the groups are analyzed using a one-way analysis of variance (ANOVA) followed by ^a, b, c, d, e^ Duncan’s multiple range test (DMRT), LSD= Least Significant Differences.*

- Nil means that no ZOI had been detected.
- AX = Amoxicillin (antibacterial standard).
- NS = Nystatin (antifungal standard).

**Table S2.** Semi-quantitative inhibition% of the biofilm formation for non-treated and treated bacterial and yeast pathogens with Co-doped TiO_2_ mesocrystals

| **Bacterial and yeast strains** | **O.D. of crystal violet stain at 570.0 nm** | | **Inhibition %** |
| --- | --- | --- | --- |
|  | **Control** | **Co-doped TiO_2_ (10.0 µg/ml)** |  |
| *Staphylococcus aureus* | 0.810^d^±0.0080 | 0.200^e^±0.0021 | 75.30% |
| *Pseudomonas aeruginosa* | 0.659^c^±0.0062 | 0.099^b^±0.0047 | 84.43% |
| *Escherichia coli* | 0.499^d^±0.0070 | 0.333^d^±0.0053 | 36.91% |
| *Klebsiellapneumoniae* | 0.555^e^±0.0025 | 0.388^a^±0.0062 | 30.03% |
| *Staphylococcus aureus* (MRSA) | 0.487^a^±0.0046 | 0.304^c^±0.0036 | 31.51% |
| *Proteus vulgaris* | 0.445^b^±0.0046 | 0.347^c^±0.0036 | 22.02% |
| *Salmonella typhi* | 0.809^f^±0.0070 | 0.179^a^±0.0053 | 77.81% |
| *Proteus mirabilis* | 0.509^c^±0.0062 | 0.109^b^±0.0047 | 78.58% |
| *Candida albicans* | 0.514^d^±0.0080 | 0.398^e^±0.0021 | 22.50% |
| *Candida tropicalic* | 0.508^d^±0.0080 | 0.390^e^±0.0021 | 23.22% |
| LSD | 0.01767 | 0.01267 | ------------ |

*Values are means ± SD (n = 3). Data within the groups are analyzed using one-way analysis of variance (ANOVA) followed by ^a, b, c, d, e, f^ Duncan’s multiple range test (DMRT), and LSD= Least Significant Differences*
